# Examining Integrated Youth Services Using the Bioecological Model: Alignments and Opportunities

**DOI:** 10.5334/ijic.4165

**Published:** 2018-11-30

**Authors:** Tanya Halsall, Ian Manion, Joanna Henderson

**Affiliations:** 1 The Royal’s Institute of Mental Health Research, CA; 2 Margaret and Wallace McCain Centre for Child, Youth and Family Mental Health Implementation, CA; 3 University of Toronto, Centre for Addiction and Mental Health (CAMH), CA; 4 Frayme, International Knowledge Translation Platform, CA; 5 University of Ottawa, School of Psychology, CA

**Keywords:** developmental systems, community-based services, youth mental health promotion, positive youth development, system transformation, holistic services

## Abstract

Integrated youth service (IYS) is a collaborative approach that brings practitioners together from across disciplines to provide comprehensive services including mental health care for youth and their families. IYS models serve as an advancement in practice as they go beyond the capacity of individual programs and services to reduce the fragmentation of care. Yet, there continue to be opportunities to expand on this perspective and promote health beyond the scope of formalized services. The bioecological model is a theoretical model that examines individual development within multiple systems of influence as well as through interactional processes between the individual and their environment. This paper provides an overview of the bioecological model and the major components of the IYS model, describing alignment and complementarity. The bioecological model provides some explanations for why IYS models may be effective and helps to provide direction to expand applied practice toward a more holistic perspective.

## Introduction

“One program, even an extraordinarily good program, cannot do it all. Young people do not grow up in programs, but in families, schools, and neighborhoods.” [Roth & Brooks-Gunn, 2003, p. 97]^1^

There is a general consensus that the current youth services system in Canada is fragmented [1,2,3,4,5]. Researchers have identified that fragmentation of services is largely an issue related to program models failing to take context into account [3] as well as placing a focus on problems [1], and the overemphasis on siloed specialized services [1,3]. The historic and current approach takes an artificially segmented perspective of developing individuals. Experts within youth development and mental health have recommended that the service system be enhanced through integration, consideration of context, and inclusion of individual voice [3,4,6,7]. Henderson and colleagues [3] warn that the system “needs transformative change that simultaneously addresses all system levels and meaningfully integrates youth and family members.” [p. 2]. Many recent advancements in system transformation have benefited significantly from innovations developed through practice-based insights and client lived experience, however there continues to be a need to examine these strategies using theoretical knowledge.

The bioecological model is a theory that was developed to better understand human development and places a focus on the agentic role of the individual as well as the multiple contextual systems involved in influencing development [8]. The bioecological model has been successfully applied within many fields to conceptualize successful holistic approaches to health and wellness promotion [9,10,11,12] and provides a potentially useful lens to support the enhancement of the youth service system.

“Integrated youth service” [IYS] is an approach that was developed in response to a lack of access and coordination of services and has been specifically recommended for application in youth populations [3]. Although eligibility criteria vary across programs, typically client ages range from 12–25. IYS is a promising practice that begins to address youth needs more holistically and places an emphasis on youth and family voice [13,14]. This article will review the major concepts related to the bioecological model and will examine how well IYS aligns with the theory. It then applies the bioecological model to identify gaps and future directions for IYS practice as well as implications for policy and program development.

## Bioecological model

The bioecological model is a theoretical frame embedded within the relational developmental systems’ (RDS) metatheoretical perspective [7]. Models within the RDS perspective place a focus on the reciprocal influence between a developing individual and a multitude of dynamic contextual levels [7]. The RDS is a central component of the Process-Relation paradigm that runs in opposition to the Cartesian scientific perspective which places an emphasis on division, duality, stability and objectivism [15,16] and perceives organisms as complicated (and thus divisible into parts for analysis) rather than complex [16]. In contrast, ontological and epistemological categories within the Process-Relation paradigm emphasize holism, dynamism, pluralism and subjectivism [16]. In line with the Process-Relation paradigm perspective, Cartesian dichotomies are perceived as false, including the division between nature-nurture, mind-body and basic versus applied science [15,16]. Overall, the Process-Relation paradigm perspective emphasizes the complexity of individual development and the importance of examining individuals holistically. This involves the inclusion of a range of individual qualities and attributes as well as the interactive relationships with contextual systems.

The bioecological model, which aims to understand development in the context of the RDS perspective, places a central focus on the four following components: 1) process, 2) person, 3) context, and 4) time [8,17,18]. *Process*, or proximal process is the most fundamental component of the model and has been defined as “progressively more complex reciprocal interaction between an active, evolving biopsychological human organism and the persons, objects, and symbols in its immediate external environment” [[Bibr B8][Bibr B17][Bibr B18]].

Within the model, the *person* is represented both as an agentic functional element involved in the process of development as well as representing individual developmental outcomes [8]. The concept of *time* relates to the temporal nature of development as the well as the historical ethos that surrounds development [8].

**Figure 1:**
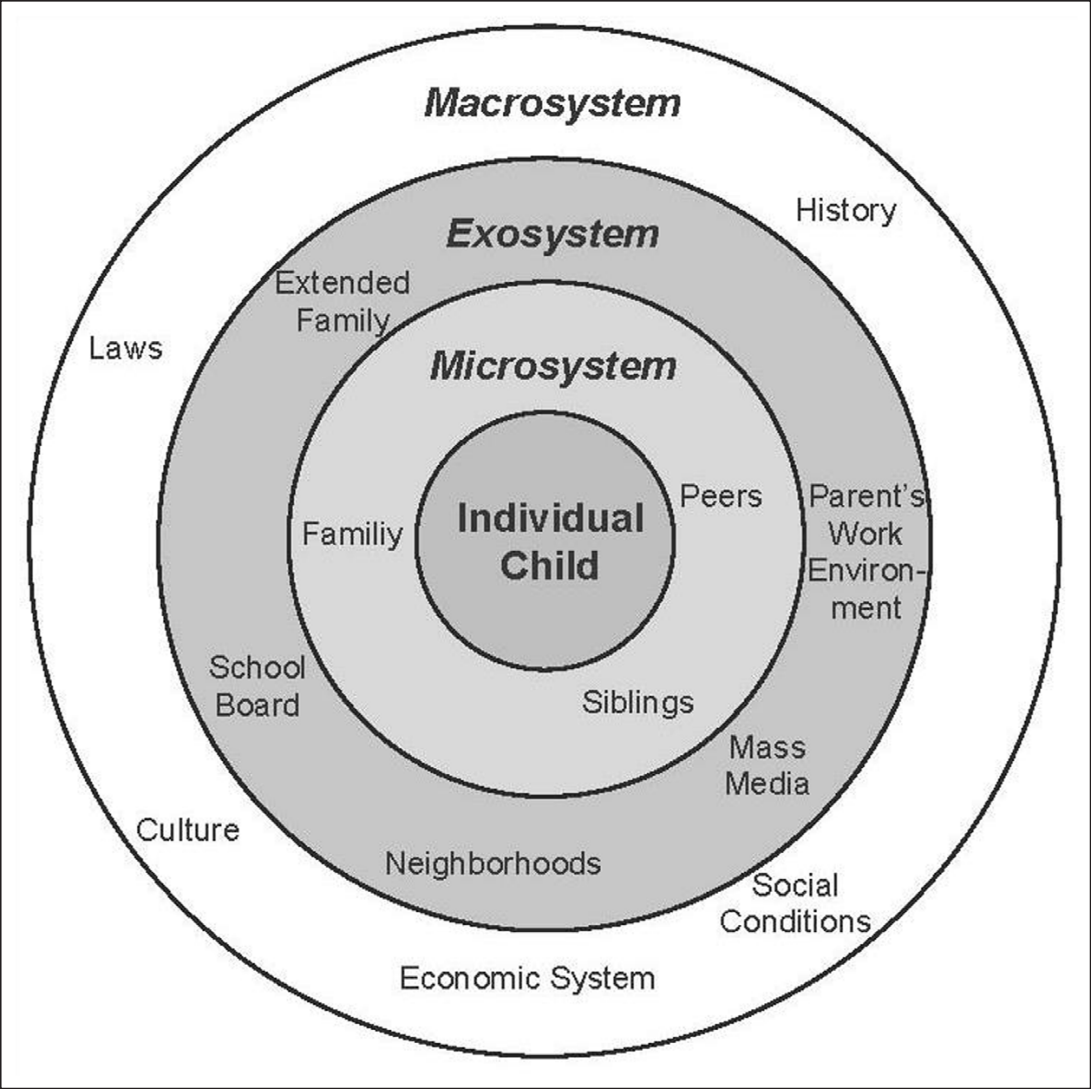
Bronfenbrenner’s Ecological Model displaying the multiple contextual levels. Original figure published in Springer [Niederer, I., Kriemler, S., Zahner, L., Bürgi, F., Ebenegger, V., Hartmann, T., … & Puder, J. J. [2009]. Influence of a lifestyle intervention in preschool children on physiological and psychological parameters [Ballabeina]: study design of a cluster randomized controlled trial. BMC Public Health, 9[1], 94.].

Finally, *context* represents the nested environmental systems that influence development and are divided into micro-, meso-, exo- and macro-systems [8,19; Figure 1]. Bronfenbrenner defines a microsystem as “a pattern of activities, roles and interpersonal relations experienced by the developing person in a given setting” [19, p. 22]. Examples of typical developmental settings would include the family home, school or places of employment. Mesosystems represent the interrelations between one or more microsystem settings. Exo-systems signify settings where the developing individual does not actively participate, but that still have an influence on their development (e.g. a school board, parental workplace or community association). The macro-system represents social and cultural norms that influence the form and nature of the other developmental systems [19,20].

**Figure 2:**
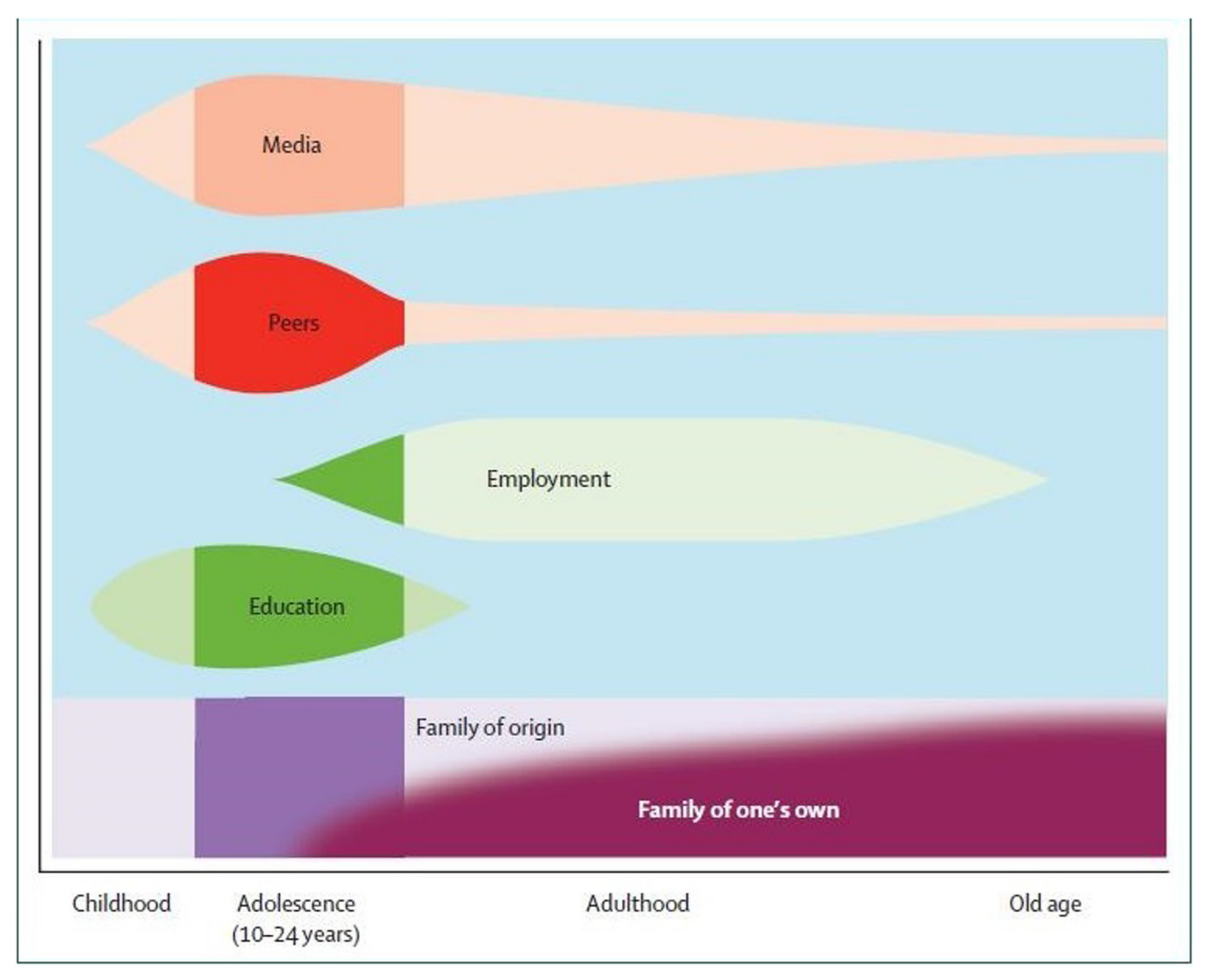
Social determinants of health as reflected across development. Family influence shifts from family of origin to the chosen family as an individual develops. During adolescence, there are multiple social determinants that influence health, including media, peers, media, education, and the workplace. Reprinted from Patton, G. C., Sawyer, S. M., Santelli, J. S., Ross, D. A., Afifi, R., Allen, N. B., … Viner, R. M. [2016]. Our future: a Lancet commission on adolescent health and wellbeing. The Lancet, 387[10036], 2423–2478 with permission from Elsevier.

Figure 2 helps to visualize the dynamic nature of multiple system influences and illustrates the interaction between the concepts of time with context. The figure depicts multiple determinants of health and the time frame in development when they exert the most influence. It demonstrates the potential ineffectiveness of conceptualizing the youth and responding services in a static perspective. As an individual develops, major influences change, and as such, intervention focus and objectives should also change. For example, based on this model, inclusion of family of origin within treatment would be highly relevant earlier in a child or youth’s development, however, later a focus on vocational supports, workplace well-being and inclusion of new spousal/family and/or significant others would be a more effective approach. Bronfenbrenner and Morris [8] highlight this consideration related to shifting significant others and their relative influence through the life span.

Previous research has successfully applied the bioecological model to a broad spectrum of disciplines, including youth development [21,22,23,24], the promotion of physical activity [11,12], school psychology [25] and mental health [10]. For example, Sallis and colleagues [12] used the bioecological model to inform the four domains of active living. They highlight that the lens of focus for the promotion of physical activity must be broadened from a strict focus on behavioural interventions that focus on exercise to multi-level interventions that include considerations related to physical environments, social norms and policy. They highlight the importance of including a range of disciplines, including professionals from public health, urban planning, transportation, recreation and policy to create comprehensive solutions to population-level engagement in active living.

Similarly, the ecological model has also been applied to inform the Systems of Care practice [10]. A system of care has been defined as: “a comprehensive spectrum of mental health and other necessary services which are organized into a coordinated network to meet the multiple and changing needs of severely emotionally disturbed children and adolescents.” [26, p. iv]. Cook and Kilmer [10] identify that typically, the Systems of Care approach focuses on the coordination of formalized services such as mental health providers and social services, as well as the supports provided to the family by these services. They note that this approach does not place enough emphasis on other community-based supports, such as peers, vocational supports and faith-based communities. They also suggest that this perspective constrains individual and family growth beyond these services. This diminishes their capacity to become positively integrated within the community within multiple avenues, such as through the development of positive relationships with neighbours, the creation of stable vocational arrangements for parents and the promotion of family engagement in positive social programs.

In another relevant application of bioecological model, Burns and colleagues [25] discuss the advantages of taking an ecological approach to school psychology that includes components such as parent training, enhanced student supervision and changes to policy. They note that using a universal ecological approach that includes positive behaviour training and early intervention strategies would be expected to decrease the number of students in need of intensive services. As such, the bioecological model can enhance school psychology practice through both breadth, by addressing system-level issues that affect a broad range of students, and depth, by better coordinating services for those who are in need [9,25].

Although it is not explicitly based on the bioecological model, the Icelandic Model of Adolescent Substance Use Prevention takes a holistic approach and has made significant population-level impacts on youth substance misuse through the enhancement of a broad range of community-based supports, including family, school and recreation [27,28]. This intervention was implemented through collaborative efforts between research, policy and practice and it involved community-based action. This included a five-year commitment of resources from municipal and federal governments, partnership development among schools and other community-based agencies, parental education and increased youth involvement in sport and extracurricular activities.

## Integrated Youth Services

Recent reviews of IYS programs identify that there is a range of models being applied internationally that each take a collaborative approach to the provision of multiple services with the objective of providing a more comprehensive response to client needs [13,14]. Some of the most common components of the services reviewed are the inclusion of mental health services, health care, and social services such as vocational assistance, educational supports and housing services. Many of the models place an emphasis on early intervention or prevention and often integrate a youth-friendly physical space, care coordination, brief therapy, peer supports, connections with primary care and technology-enhanced services in order to improve accessibility. Another common element among IYS models is the application of principles related to youth and family engagement and the inclusion of evidence-based practices. Finally, some of the approaches also apply stepped care [3,14], an assessment model that places an emphasis on matching youth with services based on the level of need.

An underlying philosophy or pillar of many of these programs is a commitment to meaningful youth engagement. This includes the development of youth-adult partnerships in order to promote youth leadership with the intention of contributing to social change [29]. This practice is also used to develop stronger insights about best practices and whether they are acceptable to youth, and also to help promote youth participation and engagement within practice settings. Youth engagement has been identified as an essential component of successful positive youth development programs [30,31] and has been associated with three main outcomes in community initiatives: 1) positive developmental impacts on the youth engaged in the initiative [32,33,34,35,36,37,38,39,40], 2) positive impacts on organizations and program operations [32,34,41], and 3) community- and system-level impacts [32,39]. In addition, through youth engagement, individuals develop life skills such as the ability to take responsibility, self-advocate and apply critical thinking. In many IYS models, youth engagement is applied at the governance-level, which facilitates youth voice having an influence on organizational and system-level strategies. Within these structures, youth are involved within advisory as well as decision-making roles and their perspectives support a shift toward procedures, programming, and practices that are more youth-friendly and better adapted to meet youth needs. The IYS approach requires collaboration among multiple service-providing organizations and recent initiatives engage youth within governance to help inform the direction of collaborative efforts and to advise strategic direction (see for example [42,43]).

## IYS through the lens of the Bioecological model

There are many ways in which IYS models align with the bioecological model. First, through purposeful design, IYS is meant to represent a breadth of contextual systems, including factors from micro-, meso-, exo- and macro-system levels. At the micro-system-level, IYS involves a range of community-based systems that directly influence youth development, including primary care, school-based programs, community-services, technology-based supports, youth-friendly hubs, peer-to-peer supports and the meaningful participation of families. Family engagement is a core value and strategy within many IYS models and involves the inclusion of family experience and perspective within practice and accounts for the supports that they contribute as family members. Since the family micro-system plays a fundamental role in influencing development [8,19], particularly in early development, consideration of this context is of significant importance. Furthermore, the inclusion of family and peer support is significant as a systemic approach of IYS, as it applies natural support systems, rather than formalized service professionals. At the meso-system level, care coordination represents the explicit consideration of the interactions between micro-systems. This includes the sharing of information, collaboration, the provision of navigators and service integration across agencies [14].

With respect to the exo-system level, IYS models require collaboration between agencies and the involvement of policy. IYS organizational partnerships require a substantial investment of time and are necessary for effective functioning at the service level. Although these networks do not have a direct influence on the developing individual, they are a necessary component to support inter-organizational collaboration and the functional delivery of seamless supports. Since these initiatives require full involvement that crosses organizational boundaries and sectors, policy is implicated within a critical role to support implementation. Adapting policies that are conducive to building relationships and that create incentives for collaboration is essential for the successful functioning of IYS. Similarly, identifying and removing policy-related barriers is equally important [e.g. mandating arbitrary or rigid age limits for service provision]. Furthermore, scaling-up individual IYS programs to create a system of care can also be considered as a critical element at the exo-system level. We re-visit the policy implications related to the ecological perspective and youth development later in the article.

At the macro-system level, cultural norms that influence youth developmental settings are implicated. Relatedly, stigma associated with mental illness has been identified as a major influence on youth access of services [44,45]. The Internet has been identified as a significant opportunity to overcome barriers to services that are related to stigma [46,47]. For example, social media presents a significant context through which to enhance health promotion campaigns and to influence healthy behaviours [48]. Furthermore, advances in artificial intelligence and technologies that support service provision are creating opportunities for low severity cases to access mental health services that are delivered online [see for example 49,50,51,52]. This is particularly useful for consumers who prefer to access services anonymously [53]. Many IYS models place a significant emphasis on technology-based services [14] and this component will be an important area for future development.

Similarly, the strong emphasis on youth engagement in IYS maps onto the “person” and “process” factors in the bioecological model. For example, through youth engagement, individual youth perspectives and needs are taken into consideration and these are used to drive treatment and practice. Youth engagement is also a proximal process, whereby youth actions are deterministic in the evolution of their treatment direction. Notably, researchers have used the bioecological model to explore various aspects of youth-adult partnerships within organizational governance structures [23]. They conceptualize the organizational context where youth are engaged as micro-systems and the external organizational contexts, such as the Board of Directors as exo-systems.

The last major component of the bioecological model is time [8]. This component emphasizes temporal qualities of an individual’s development and highlights the notion that a holistic consideration of an individual must extend into their past and future. Currently, one of IYS models’ contributions to practice is that they consider service needs across development, allow for young people to move in and out of service seamlessly over time as their needs change, and do not insert mandated, and often unsupported, service changes at important developmental transition points, such as at the transition to emerging adulthood around age 18. Instead, the model spans these transition points and captures individual needs more holistically as it reaches beyond services and program boundaries in order to support the individual as they move into adulthood. IYS models are also unique in that they place an emphasis on youth, including emerging adults, as well as co-creation with youth, co-location of services within a youth-friendly setting, and the enhancement of immediate access.

## Future Directions

Based on the above considerations related to the current conceptualization of the IYS model and the theoretical frame that the bioecological model can provide, we will describe implications for future practice, policy and research within youth mental health promotion.

## Recommendations for IYS practice

The bioecological model can inform several major directions that could be applied to enhancing IYS practice going forward. The bioecological model and other developmental systems theories provided the foundation for the development of the positive youth development (PYD) approach and it may be beneficial to use this framework when examining IYS programming. PYD is a holistic strengths-based approach that places an emphasis on positive developmental impacts and influences that lead to successful development [54]. This perspective is contrasted with deficit or symptom-based approaches [55] and related programming often emphasizes the development of leadership, positive relationships and life skills [30].

Despite placing an emphasis on holistic approaches, with the exception of a few examples of more comprehensive school-based programs [6,56] most PYD program models are isolated services applied within sport, extra-curricular and school-based contexts [see [57,58,59]. Recognizing this shortcoming, the IYS model represents a possible advancement in PYD practice whereby specialized community services that are better equipped to support a range of individual needs and strengths are brought together.

Conversely, the bioecological model and the PYD framework can be used to enhance current IYS models through an increased focus on promotion and strengths-based strategies. For example, although IYS takes a more holistic approach to meeting the needs of the developing individual, many models place a focus on treatments post-diagnosis. Accessing youth earlier in development may prevent some individuals from reaching a clinical level where they would receive a diagnosis. Taking this approach would implicate schools as a major target for the implementation of universal promotion and prevention efforts. Other researchers have highlighted that it is critical to consider the inclusion of schools [60] and educational objectives within youth mental health promotion initiatives [6]. For example, Burns and colleagues [25] have advocated for the use of school-based system interventions that apply universal screening, positive behavioural supports and small group interventions [9,25]. In particular, schools may be an important context in which to embed peer and family support services from IYS. Peer and family support services provided in schools may help to overcome access issues related to stigma. For example, receiving support from successful peer role models that are coping with similar symptoms and presenting issues may help youth to overcome barriers related to stigma and also help to alleviate wait times for professional services.

This shift also implicates connections with more universal interventions that can be accessed by the general population and the inclusion of recreation and other community-based child and youth programs within IYS models. In Canada, universal health promotion interventions have mainly been applied to early childhood development [5], however these efforts must be extended through the developmental stages. For example, based on the bioecological model, Bronfenbrenner and Morris argue that “as children grow older, their developmental capacities increase both in level and range; therefore, to continue to be effective, the corresponding proximal processes must also become more extensive and complex to provide for the future realization of evolving potentials” [8 p. 798]. This suggests that universal strategies that do not extend beyond the early developmental years will not support the continued advancement of individual capacity through the next stages of maturity.

Transitions to adult services and supports should be seamless [4], and supports for transitional age youth are a major focus in IYS. However, youth-based interventions should take factors into consideration that will have an enduring impact throughout an individual’s lifespan. For example, the bioecological model emphasizes the critical role of social relationships, and family/significant others in particular, in human development [8]. Figure 2, illustrates the shift in influence from the family of origin to the “family of one’s own” that begins during emerging adulthood and continues into old age. A “family of one’s own” represents the new family relationships that are selected and cultivated by an individual as they become independent adults. This demonstrates that new family/spousal investments or significant others are likely to play a critical role in interventions for emerging adults as they are the key system of influence through to the end of the individual life span. Where indicated, interventions should place a focus on enhancing relationships with significant others and strengthening family connections. Many IYS models include a flexible definition of family and their engagement strategies involve both natural families as well as significant others. This practice should be expanded within other IYS models.

Some IYS approaches also place a focus on providing vocational supports, and this practice should be applied more broadly. As indicated in Figure 2, employment represents another influential factor that extends into later adulthood. A recent report from the Expert Panel on Youth Employment at Employment and Social Development Canada [61] recommends that employment initiatives take a collaborative, ecological perspective that engages youth in order to promote youth involvement in the Canadian workforce. They also recommend applying innovative and relevant mentorship programs, expanding the government youth employment program, engaging youth in re-vamping job search technology and engaging inter-sectoral partners in hiring strategies.

Some school programs offer a significant opportunity for youth to explore areas of interest for career development as well as to offer connections to employers for talent development and recruitment. For example, the Specialist High Skills Major program is an initiative led by the Ontario Ministry of Education designed to allow “students to focus their learning on a specific economic sector while meeting the requirements to graduate from secondary school. It also assists in their transition after graduation to apprenticeship training, college, university or the workplace” [62]. These programs offer specializations in a range of sectors and allow students to earn educational credits while also exploring career pathways and relevant skills. Programs like this would be a useful complement to other IYS services that would provide positive individual and economic benefits for an extended period of time. We recommend that IYS programs include assessments that examine aptitudes and career interest and actively seek out and partner with local programs that offer similar career development opportunities.

In terms of identifying the appropriate level of treatment to offer, many IYS models utilize a stepped care approach [3,14]. This strategy organizes treatment alternatives hierarchically by intensity and care decision-making is based on specific criteria [63]. Additionally, stepped care models have been described as prioritizing lower intensity treatment and then increasing intervention intensity “stepping up” if the individual does not respond positively [64]. Initial stepped care models emphasized diagnostic criteria and utilize assessments that focus on symptoms, although more recent efforts have moved away from this approach. Considering individuals holistically, we would recommend that IYS models incorporate strengths-based measures within their stepped care approaches and provide referrals to activities and programs that promote youth involvement in individual interests and enhance skills. In Iceland, within the Model of Adolescent Substance Use Prevention measurement strategy, researchers collected information about family and peer relationships, well-being and participation in extra-curricular activities within their assessments [27]. Using this information, their intervention involved a system-level approach that included parental education, increasing youth involvement in sport and extracurricular activities, and the promotion of partnership between local schools and other community-based agencies [28]. Data collected after the implementation of this strategy demonstrated that youth time spent with parents and time spent in organized sport had increased. In addition, the proportion of youth getting drunk on a regular basis was reduced by over 50% and the proportion of youth who had used hashish by over 60%. IYS models that incorporate strengths-based assessments may be able to take advantage of referring to local programs and activities that could develop strengths and interests of participating youth.

Applying an assessment strategy that examines both strengths and challenges creates a more holistic representation of the individual and other researchers have advocated for the use of strengths-based assessments in combination with assessment of presenting issues and to use these findings to inform intervention strategy [9]. This methodology would also be useful to avoid stigmatization as well as creating social capital based on strengths and interest. For example, when assessment findings are used to direct the youth to a group or activity based on a shared area of interest, stigma would not present a challenge for participation and peers would be brought together based on their shared interests and skills rather than deficits. In addition, this provides youth with the advantage of benefitting from a more positive reference group, developing a sense of belonging within a positive social setting and may also strengthen longevity of their involvement and development of skills and aptitudes.

Finally, social media and the online environment represents a new cross-level context that spans the micro-system to the macro-system. This virtual context has a pervasive influence on modern youth and some research has begun to demonstrate that social media may create a significant threat to the well-being of youth and young adults [65,66,67]. Others, however, have identified the potential of technology and social media to promote wellbeing [52,53,68]. Recognizing this system of influence on youth development, it is essential to consider the potential for emerging technology to provide solutions for wellness promotion, stigma reduction, mental health literacy and mental health care [48]. This is particularly relevant for remote jurisdictions where access to traditional services and supports is a real challenge. Web-based IYS services offer virtual supports such as tools to enhance mental health literacy, stories about lived experience, online counselling and information regarding how to find help. As a complementary system, researchers should continue to explore and evaluate web-based IYS services in context [e.g., Beyond Blue, E-Foundry, E-Headspace] and IYS programs should place a significant focus on leveraging social media to promote youth mental health and development through the use of social marketing strategies. It may also be useful for IYS programs to partner with technological organizations that have already achieved deep penetration of the target audience (e.g. Facebook currently has 2 billion active monthly users [69]). This would increase reach to existing networks and may support the development of algorithms that create healthy choice architectures for a broad range of users [48].

## Recommendations for policy

Important policy implications stem from the roles macro-and exo-systems play in influencing social norms and regulating behaviour. One of the major contributions of the bioecological model is that it places a comprehensive focus on the full range of developmental stages and considerations for application to policy and program contexts [8, p. 794]. Furthermore, the World Health Organizations stresses the critical importance that child and youth mental health policy support interventions across the range of needs from promotion to complex intervention and recommends that initiatives assign financial contributions according to the population size implicated [70]. This identifies the highest investment at the level of health promotion and self-care. In addition, the WHO recommends that multiple sectors and service systems must be involved within a cohesive policy direction in order to reduce fragmentation of services.

Accordingly, there are many recommendations for policy development related to youth mental health promotion and IYS that can be developed based on the predictions of the bioecological model. Sallis and colleagues’ [12] multi-level interventions applied the bioecological model to the promotion of physical activity. This work demonstrates that shifting the focus from targeting individual exercise behaviours to applying multi-level interventions, that include municipal planning, transportation infrastructure, workplace and educational norms can have much broader-level population health impacts. Furthermore, it highlights the importance of taking a holistic perspective of context and how powerful this can be when directed to policy reform. Applying this perspective to IYS practice, this work highlights the important role that policy plays in enhancing collaboration between organizations. Furthermore, this approach indicates an analogous shift in perspective related with youth services whereby policy targets should consider, not only behavioural interventions and formalized programming for youth, but also contexts that influence children and youth on a daily basis, such as within schools, communities, and more recently, the online environment.

For example, policy plays an important role in supporting collaboration among organizations that are implementing IYS programs. Currently, there is a range of obstacles that inhibit the integration of services through inter-organizational collaboration, including bureaucratic obstacles, organizational change aversion, hierarchies of influence among stakeholders and cultures of competition [71]. One avenue to support collaboration and system transformation is through intermediary organizations that promote collaboration, knowledge exchange, capacity-building and policy-level strategies. In Ontario, the Centre for Addiction and Mental Health and the Ontario Centre of Excellence for Child and Youth Mental Health are both uniquely positioned to promote these system changes. Also based in Canada, the Frayme/Cadre network is an international network designed to enhance global youth well-being through the provision of knowledge exchange and implementation supports regarding youth-centered IYS. Investments in organizations such as these help to support the collaborative partnership among agencies and facilitate the work required in implementing IYS.

The following political strategies that influence the macro-system level have the potential to promote access to IYS programs as well as diminish the need for more intensive services within IYS. For example, researchers have emphasized the importance of policy-level limit-setting so that youth do not have the opportunity to engage in risky behaviours [1] and practice-based evidence has demonstrated that policies that regulate negative behaviour can have broad population impacts. For example, in Canada, policies that enforce smoke-free environments have been found to be effective in reducing smoking behaviour and negative impacts on health in Canada [72,73] and these strategies are endorsed by the WHO [74]. Similarly, in Iceland the establishment of curfews have been effective in the reduction of substance use in youth [27]. These policies decrease the amount of time youth spent in unstructured activities and enhance familial support [28].

Turning attention to the online environment, in Canada, 17% of children and youth between the ages of 15 and 29 have been cyberbullied or cyberstalked sometime in the previous five years [75]. Furthermore, researchers have identified that many youth are now presenting with internet addiction [76,77]. Recognizing the increasing risks related with the internet and social media, it might also be useful to create and enforce policy that discourages smart phone use for children under a specific age and to regulate organizations that develop online environments for youth so that they encourage positive behaviours and support well-being and development.

It may be possible to create incentives or regulations that support the creation and adaptation of these environments through policy. Policy-makers must recognize that social media and other web-based environments are significant contexts that should be adapted to support health promotion for youth [48]. Applying nudge theory, the online environment can be visualized as a choice architecture that can have a pervasive and significant influence on youth’s day-to-day behaviour and overall wellbeing [48]. It may be helpful to support the uptake of algorithms that enhance supportive social interaction and obstruct exchanges that might be indicative of cyber-bullying within popular social media applications [48]. Furthermore, evidence-based e-services provided as an adjunct to IYS programs may play an important role in expanding online environments that promote health and wellbeing for youth. Policy-makers and funders should promote the uptake of these services so that IYS programs may expand reach and enhance access.

## Recommendations for research

In terms of research generated, the nested contextual systems of the bioecological model have been more influential in stimulating research than the other three components and investigators have suggested that new examinations take account of the more recent developments to the model that include the concepts of person, process, context and time [20]. In applying the bioecological model to the examination of IYS practice, the process of youth engagement within IYS represents a unique opportunity for empirical exploration of influences on personal development as well as context. As stated earlier, youth engagement strategies can be described as a unique form of proximal process and present a significant phenomenon to examine using the bioecological model lens. For example, since youth engagement is now being applied to IYS governance at the system-level, studies can examine specific skills and assets developed within the youth engaged in advocacy efforts as well as how their input influences the direction of organizational strategies and impacts on system-level practice. Changes to system-level practice may also have relevance to shifting cultural and social norms which is implicated within Bronfenbrenner’s fifth principle which “asserts that changes over time in the four defining properties of the bioecological model are not only products but also producers of historical change” [8 p. 822]. If social norms and concepts related to youth are affected by system-level shifts developed through IYS initiatives, this may be a reflection of how individual agency can have an impact on societal development.

As part of this examination, Bronfenbrenner and Morris [8] also highlight the importance of examining both sides of a reciprocal interaction. Since youth engagement at the governance level implicates youth-adult partnerships, both of these perspectives should be captured within future research. In addition, there continues to be a need for more research that examines youth engagement and the influence on organizational and system impacts [39,78,79,80] and there is a need for more research that examines youth engagement as a process, particularly with respect to influences associated with external organizational structures [80], implementation [79,81], representative youth diversity [82] and development of social capital [82,83].

It would also be beneficial to examine comparisons between typical IYS practice and models that adopt some of the aforementioned recommendations as comparators. For example, it would be informative to investigate the effects of implementing an IYS model within a school as compared with another context. Identifying what components are minimally required within IYS to maximize benefits would be helpful to inform policy and practice.

Future research of IYS models should apply mixed methods that include a qualitative approach to capture contextual influences and system level adaptations. Futch Ehrlich [84] has emphasized the importance of applying qualitative strategies to examine youth development as it facilitates the examination of contextual interactions, process and supports the inclusion of youth through narrative. Furthermore, Bronfenbrenner [19] has highlighted the importance of empirically capturing the *perceptions* of experience within developmental contexts and qualitative methods are well–designed to explore subjective experience. There are also new methodologies that have been designed to examine the complexities of collaborative and innovative initiatives such as developmental evaluation [85,86] and principles-focused evaluation [87] that incorporate qualitative strategies that would be useful to apply within these research approaches.

Finally, social media represents a significant tool for IYS models to use in engaging youth. It also represents a novel context that spans the micro-system through to the macro-system-level. Future studies that examine SM both as a tool to promote IYS as well as a way to capture current youth perspectives to inform IYS practice would be a productive future research direction. Furthermore, the collection of data through SM may represent an independent research method.

## Conclusion

This article described the major components of IYS and the bioecological model and examined areas of alignment. It also used the bioecological model to identify gaps and future directions for IYS practice as well as implications for policy, practice and future research. We argue that the IYS model is a significant advancement in practice with regard to providing comprehensive supports to youth and that using the bioecological model, practice and policy can be expanded to better support the holistic promotion of youth well-being.

Recently, the Lancet commission brought together global leaders in the field to report on the current state of youth health and wellbeing [88]. They argued that “we have come to new understandings of adolescence as a critical phase in life for achieving human potential… This generation of adolescents and young adults can transform all of our futures; there is no more pressing task in global health than ensuring they have the resources to do so” [p. 2423–2425]. Investment in youth should not be perceived as solely a strategy for health care cost savings. By applying ecological perspectives to inform applied approaches for youth, we can develop holistic strategies that invest in our collective future and enhance the health of our society as a whole.

## Footnotes

Roth, JL & Brooks-Gunn, J. What exactly is a youth development program? Answers from research and practice. Applied Developmental Science, 2003; 7(2), 94–111.
